# Trajectory of the time course for LVH regression and remodeling imparted by aortic valve replacement for severe aortic stenosis; a cardiovascular MRI study sponsored by the American Heart Association

**DOI:** 10.1186/1532-429X-11-S1-P207

**Published:** 2009-01-28

**Authors:** Robert WW Biederman, James A Magovern, Saundra B Grant, Ronald B Williams, June A Yamrozik, Diane A Vido, Vikas K Rathi, Mark Doyle

**Affiliations:** Allegheny General Hospital, The Gerald McGinnis Cardiovascular Institute, Pittsburgh, PA USA

**Keywords:** Aortic Stenosis, Aortic Valve Replacement, Reverse Remodel, Severe Aortic Stenosis, Favorable Alteration

## Background

In patients with severe aortic stenosis (AS), long-term data tracking surgically induced beneficial effects of afterload reduction via aortic valve replacement (AVR) on reverse LV remodeling are not available. Echocardiographic data is available short-term, but in limited fashion beyond one year. Cardiovascular MRI (CMR) offers the ability to track discrete changes in LV metrics with small numbers but high accuracy due to its inherent high spatial resolution and low variability.

## Hypothesis

We hypothesize that progressive changes following AVR are detectable by CMR and changes in LV structure and function, triggered by AVR, continue to favorably improve over an extended period following AVR.

## Methods

Ten pts (67 ± 12 yrs, 6 female) with severe, but compensated, AS underwent CMR pre AVR and post AVR at 6 ± 2 months, 1 year ± 2 month, and up to 4 yearrs ± 5 month. LV mass index (LVMI), LV geometry, volumetrics and EF were measured (GE, EXCITE 1.5 T, Milwaukee, WI). A Kruskall-Wallis one-way ANOVA was performed.

## Results

**Figure 1:**
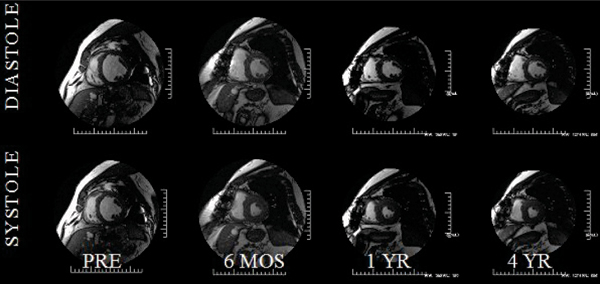
Figure 1

Despite advanced but mostly compensated AS ((peak and mean gradient by CMR (67 ± 21 and 43 ± 10 mmHg, respectively) and by Echocardiography (72 ± 23 and 44 ± 11, resepctively) (p = NS for all)), all 10 pts survived AVR and underwent CMR at up to a 4-year time point (40 time points). LVMI markedly decreased at 6 months (157 ± 42 to 134 ± 32 g/m^2^, p < 0.005), continuing to gently trend downwards at 4 yrs (127 ± 32 g/m2). Similarly, EF increased pre to post AVR (55 ± 22 to 65 ± 11%, (p < 0.05)) and continued trending upward, remaining stable at years 1–4 (66 ± 11 vs. 65 ± 9%). LVEDV index, initially high pre AVR, normalized post AVR (83 ± 30 to 68 ± 11 ml/m2, p < 0.05), trending even lower by year 4 (66 ± 10 ml/m^2^). LV stroke volume increased rapidly from pre to post AVR (40 ± 11 to 44 ± 7 ml) continuing to gradually increase at 4 yrs (49 ± 14 ml, p = 0.3). Most importantly, LVMI/volume, a 3D measure of LV geometry, remained unchanged initially but over 4 years markedly improved (1.07 ± 0.2 to 0.94 ± 0.24, p < 0.05), all paralleling improvements in NYHA (3.2 ± 1.0 to 1.5 ± 1.1, p < 0.05). Figure 1.

## Conclusion

After the initial beneficial effects imparted by AVR in severe AS patients, there are, as expected, marked improvements in LV reverse remodeling. We have shown, via CMR, that surgically induced benefits to LV structure and function, including favorable alterations in LV geometry, are durable and, unexpectedly, show continued improvement extending through 4 years post-AVR concordant with sustained improved clinical status. The pattern of this improvement is, however, previously unrecognized. Namely, a steep trajectory of improvement early in which fully 75% of the effect that was to be present by year 4 is completed within the first 6 months. Afterwards, the slope of LV remodeling is much less steep and appears to become asymptotic.

